# Movement and storage of nematocysts across development in the nudibranch *Berghia stephanieae* (Valdés, 2005)

**DOI:** 10.1186/s12983-022-00460-1

**Published:** 2022-04-18

**Authors:** Jessica A. Goodheart, Vanessa Barone, Deirdre C. Lyons

**Affiliations:** Scripps Institution of Oceanography, University of California, San Diego, La Jolla, CA 92037 USA

**Keywords:** Nudibranch gastropods, Intracellular sequestration, Nematocysts, Cell and particle recognition, Juvenile development

## Abstract

**Background:**

Intracellular sequestration requires specialized cellular and molecular mechanisms allowing a predator to retain and use specific organelles that once belonged to its prey. Little is known about how common cellular mechanisms, like phagocytosis, can be modified to selectively internalize and store foreign structures. One form of defensive sequestration involves animals that sequester stinging organelles (nematocysts) from their cnidarian prey. While it has been hypothesized that nematocysts are identified by specialized phagocytic cells for internalization and storage, little is known about the cellular and developmental mechanisms of this process in any metazoan lineage. This knowledge gap is mainly due to a lack of genetically tractable model systems among predators and their cnidarian prey.

**Results:**

Here, we introduce the nudibranch *Berghia stephanieae* as a model system to investigate the cell, developmental, and physiological features of nematocyst sequestration selectivity. We first show that *B. stephanieae*, which feeds on *Exaiptasia diaphana*, selectively sequesters nematocysts over other *E. diaphana* tissues found in their digestive gland. Using confocal microscopy, we document that nematocyst sequestration begins shortly after feeding and prior to the formation of the appendages (cerata) where the organ responsible for sequestration (the cnidosac) resides in adults. This finding is inconsistent with previous studies that place the formation of the cnidosac after cerata emerge. Our results also show, via live imaging assays, that both nematocysts and dinoflagellates can enter the nascent cnidosac structure. This result indicates that selectivity for nematocysts occurs inside the cnidosac in *B. stephanieae*, likely in the cnidophage cells themselves.

**Conclusions:**

Our work highlights the utility of *B. stephanieae* for future research, because: (1) this species can be cultured in the laboratory, which provides access to all developmental stages, and (2) the transparency of early juveniles makes imaging techniques (and therefore cell and molecular assays) feasible. Our results pave the way for future studies using live imaging and targeted gene editing to identify the molecular mechanisms involved in nematocyst sequestration. Further studies of nematocyst sequestration in *B. stephanieae* will also allow us to investigate how common cellular mechanisms like phagocytosis can be modified to selectively internalize and store foreign structures.

**Supplementary Information:**

The online version contains supplementary material available at 10.1186/s12983-022-00460-1.

## Background

The intracellular sequestration (theft and storage) of structures or cells can be an incredibly useful tool for understanding how common cellular mechanisms, like phagocytosis, can be modified to selectively internalize and store foreign structures. Intracellular sequestration is widespread across Metazoa, with functions primarily related to metabolism (e.g., kleptoplasty [1, 2] and cnidarian-dinoflagellate endosymbiosis [3]) or defense (e.g., nematocyst sequestration [4, 5]). Most cellular and molecular investigations have centered on cnidarian-dinoflagellate endosymbiosis [6], a metabolic system. Those studies led to insights into how oxidative environment regulation is involved in the molecular co-evolution of symbiosis [7] and how we might use knowledge of these symbioses to assist in building coral reef resilience [8]. However, selectivity for particular symbionts is more flexible than originally thought [9] and relies on the specificity of both the symbiont and host [10]. In some cnidarians, cells that sequester symbionts may also be less selective at the initiation of phagocytosis [11]. These challenges make it more difficult to identify how the early stages of phagocytosis have been modified in cnidarian-dinoflagellate systems. In defensive sequestration systems, however, it is much easier to investigate the cell and molecular specificity of sequestration, since defensive structures and chemicals have been stripped from their original cell.

One such defensive form of sequestration, called nematocyst sequestration, involves animals that sequester stinging organelles (nematocysts) from their cnidarian prey [4, 5]. In nematocyst sequestration, there is a unique opportunity to investigate the mechanisms of selectivity for a specific type of organelle, which has been separated from its original cell [12–14] and thus does not possess membrane-associated antigens often used by phagocytes to target cells or cell fragments for engulfment [15, 16]. This type of sequestration contrasts with many others, which usually involve the internalization of whole cells [3] or of small chemicals that do not require internalization via phagocytosis [17–19]. In most groups that sequester nematocysts, it is hypothesized that these organelles are identified for internalization and storage by specialized phagocytic cells [4, 5]. Furthermore, in some species selectivity for certain nematocyst types has also been described (e.g., nudibranch gastropods [20, 21]), which has been shown to change based on external cues like predator presence. However, we know little about how these specialized phagocytic cells identify and select for nematocysts. This gap in knowledge is largely due to the fact that there are currently no laboratory models for describing the cell and molecular processes involved in nematocyst sequestration, though some experiments have been performed in both nudibranchs [22] and flatworms [23, 24].

Here, we introduce the emerging model nudibranch, *Berghia stephanieae*, as a research organism to investigate the cell and developmental features of nematocyst sequestration selectivity. *B. stephanieae* is a nudibranch gastropod, a member of the most well-studied clade of nematocyst sequestering animals, called Aeolidida (= Aeolidoidea) [25–27]*. B. stephanieae* feeds on the cnidarian *Exaiptasia diaphana*, an anemone used to investigate cnidarian-dinoflagellate endosymbiosis [28], and is known to sequester nematocysts from *E. diaphana* [25, 29]. In nudibranchs, morphology of sequestering tissues [25, 29], function of these stolen nematocysts [20, 30–33], and some physiology [22] have been explored. Like other nudibranchs, *B. stephanieae* sequesters nematocysts in a structure called the cnidosac that is found at the tips of finger-like dorsal projections called cerata [25]. Within the cnidosac, special cells called cnidophages store sequestered nematocysts for long-term use [5, 25]. Although it is known that cnidophages are where nematocysts are phagocytosed and stored [5, 25], previous research has not provided evidence that these cells are where selectivity for nematocysts occurs. One alternative hypothesis is that nematocysts are the only structures to reach the cnidophages, and therefore these cells phagocytose only the structures with which they interact. By studying *B. stephanieae*, we take advantage of the experimental tractability and transparency of this species to investigate the cellular and molecular mechanisms that allow cnidophages to selectively sequester nematocysts.

In this paper, we make use of early developmental timepoints to investigate when and where nematocyst sequestration selectivity occurs in *B. stephanieae*. It is necessary to investigate this process in earlier stages because adult cerata are pigmented and difficult to image. We first describe the adult cnidosac histologically in *B. stephanieae* as a baseline for our understanding of development. We also confirm the selectivity of nematocysts in the *B. stephanieae* cnidosac and identify at least three nematocyst types that are found sequestered inside the nudibranch. We then show that the cnidosac develops early in juvenile development, within 2–4 days post initial feeding, and that the juvenile cnidosac includes all of the standard hallmarks of the adult cnidosac. Finally, we take advantage of the transparency of the juvenile cnidosacs to time-lapse image nematocysts (and other food particles) moving through the digestive tract into the cnidosac. This work provides a starting point for assessing the rate at which nematocysts may be sequestered by *B. stephanieae*, and supports the hypothesis that cnidophages (inside the cnidosac) are the site of nematocyst selectivity.

## Results

### Adult cnidosac morphology

**Fig. 1:**
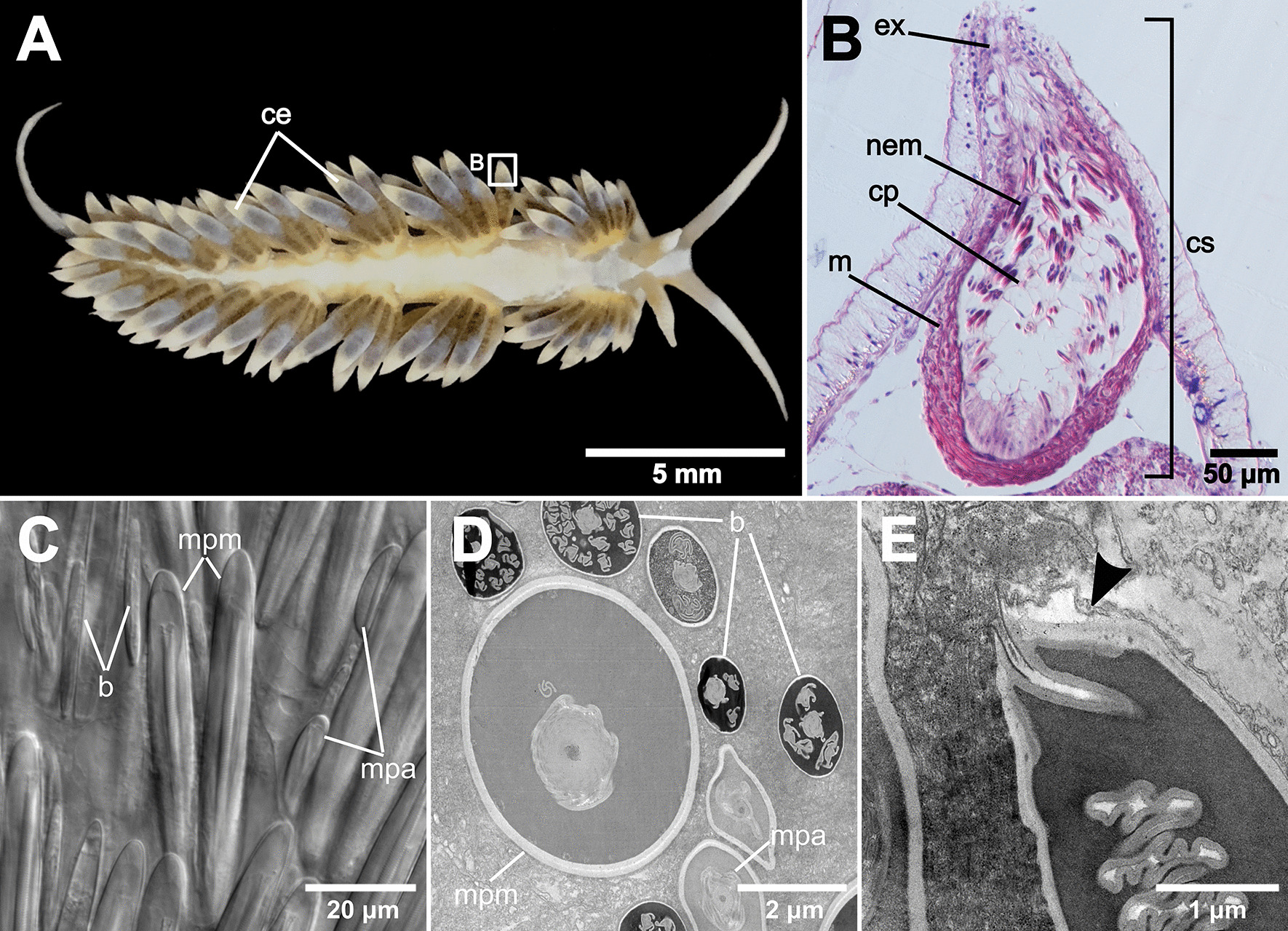
*Berghia stephanieae* adult cerata, cnidosac, and select images of sequestered nematocysts. **A** Adult animal with many cerata found on the dorsum (Photo credit: Park Masterson). Box indicates the tip of the ceras, where the cnidosac is found. **B** Histological section of an adult *B. stephanieae* cnidosac (SIO-CIC M18637), including the cnidophages and sequestered nematocysts. Multiple nematocyst types are sequestered inside the adult *B. stephanieae* cnidosac, which are shown in (**C**) Differential Interference Contrast (DIC) imaging showing the shapes and sizes of various sequestered nematocyst types, and **D** Transmission Electron Microscopy (TEM) which shows the differences in internal morphology among nematocyst types. **E** The vacuole double membrane (arrow) surrounding individual nematocysts is also visible with TEM. Abbreviations: b, basitrich nematocysts; ce, cerata; cp, cnidophages; cs, cnidosac; ex, exit; m, musculature; mpm, large microbasic p-mastigophore nematocysts; mpa, microbasic p-amastigophore nematocysts; nem, sequestered nematocysts

**Fig. 2:**
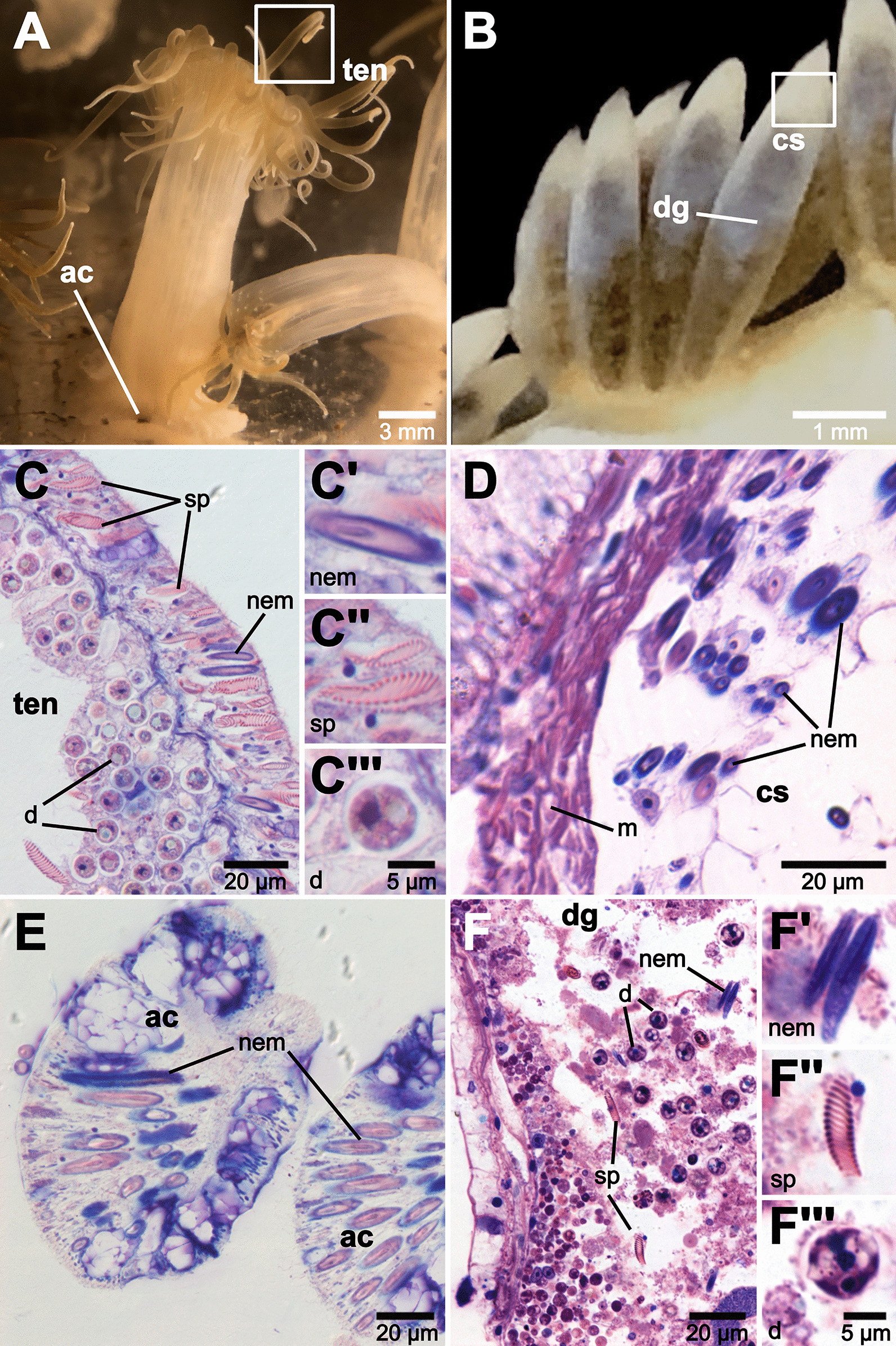
Histological sections of tissues from **A** *Exaiptasia diaphana* and **B** *Berghia stephanieae.* Only nematocysts (purple capsules around pink or purple shafts) from the anemone tissue are sequestered inside cnidosacs, whereas spirocysts (pink spiral with unstained capsules) and dinoflagellates remain in the digestive gland in *B. stephanieae*. *E. diaphana* tissues (cross section) include **C** tentacles (SIO-BIC Co3615) and (**E**) acontia (SIO-BIC Co3616). *B. stephanieae* tissues (longitudinal section) include (**D**) the cnidosac (SIO-CIC M18637) and (**F**) digestive gland (SIO-BIC M18638). Panels C and F show close-up images of nematocysts (**C’**,**F’**), spirocysts (**C’’**,**F’’**), and dinoflagellates (**C’’’**, **F’’’**). Abbreviations: ac, acontia; cs, cnidosac, d, dinoflagellates, dg, digestive gland; m, musculature; nem, nematocyst(s); sp, spirocyst(s); ten, tentacles

As previously described [25], adult *Berghia stephanieae* possess a single cnidosac at the distal end of each ceras (Fig. 1A), which is connected to the digestive gland via a short channel (Additional file 1: Figure S1). The cnidosac has a multi-layered, thick musculature (Fig. 1B). *B. stephanieae* does not appear to have a permanent pore at the distal end of the cnidosac, meaning we see no constriction of the musculature nor a clear epithelial lining towards the proximal end of the ceras (Fig. 1B). The lack of a pore in this species is consistent with the description provided by Goodheart et al. [25]. Inside the cnidosac, multiple sequestered nematocysts (also known as kleptocnides) are clustered within cnidophage cells (Fig. 1B, Additional file 1: Figure S1). The cnidophages contain different types of nematocysts obtained from their prey (*Exaiptasia diaphana*), which are often present in the same cnidosac at the same time (Fig. 1C, D). Nematocysts identified inside the cnidosac include basitrichous isorhizas (basitrichs), microbasic p-mastigophores (the largest nematocysts solely found in the acontia of *Exaiptasia diaphana*), and microbasic p-amastigophores of various sizes [34, 35] (Fig. 1C, D). We identified: (1) large microbasic p-mastigophores by their distinctive v-shaped notch and large capsule size (> 50 μm in length; Fig. 1C and Additional file 1: Figure S2A), and by their shaft, which has larger spines toward the tip of the shaft and ends in a short tubule (Additional file 1: Figure S3A); (2) small and medium sized (Fig. 1C) microbasic p-amastigophores based on the distinctive v-shaped notch on the shaft (Additional file 1: Figures S2B-C) and their short shaft with larger spines towards the coned tip (Additional file 1: Figure S3B-C); and (3) basitrichs based on their narrow, elongate shape (Fig. 1C and Additional file 1: Figure S2D-E), and the thin shaft with large spines at the base and smaller spines toward the tips (Additional file 1: Figure S3D) or consistently small spines along the entire shaft (Additional file 1: Figure S3E). In histological sections, spirocysts were differentiated from nematocysts (purple-stained capsules) by their unstained capsule and characteristic strongly coiled tubule that stains pink to pinkish purple [35] (Fig. 2C). In *E. diaphana*, we identified spirocysts in the tentacles (Fig. 2C), but not in the acontia (Fig. 2E). In *B. stephanieae*, we only found spirocysts in the digestive gland (Fig. 2D), but not inside the cnidosac (Fig. 2F). In TEM images, we noted that sequestered nematocysts are separately bound in individual vacuoles (Fig. 1E), but what causes the apparent bundling of these nematocysts (i.e., keeps nematocysts close together) within each cnidophage (Fig. 1B) is still unknown.

### Cnidosac development

**Fig. 3:**
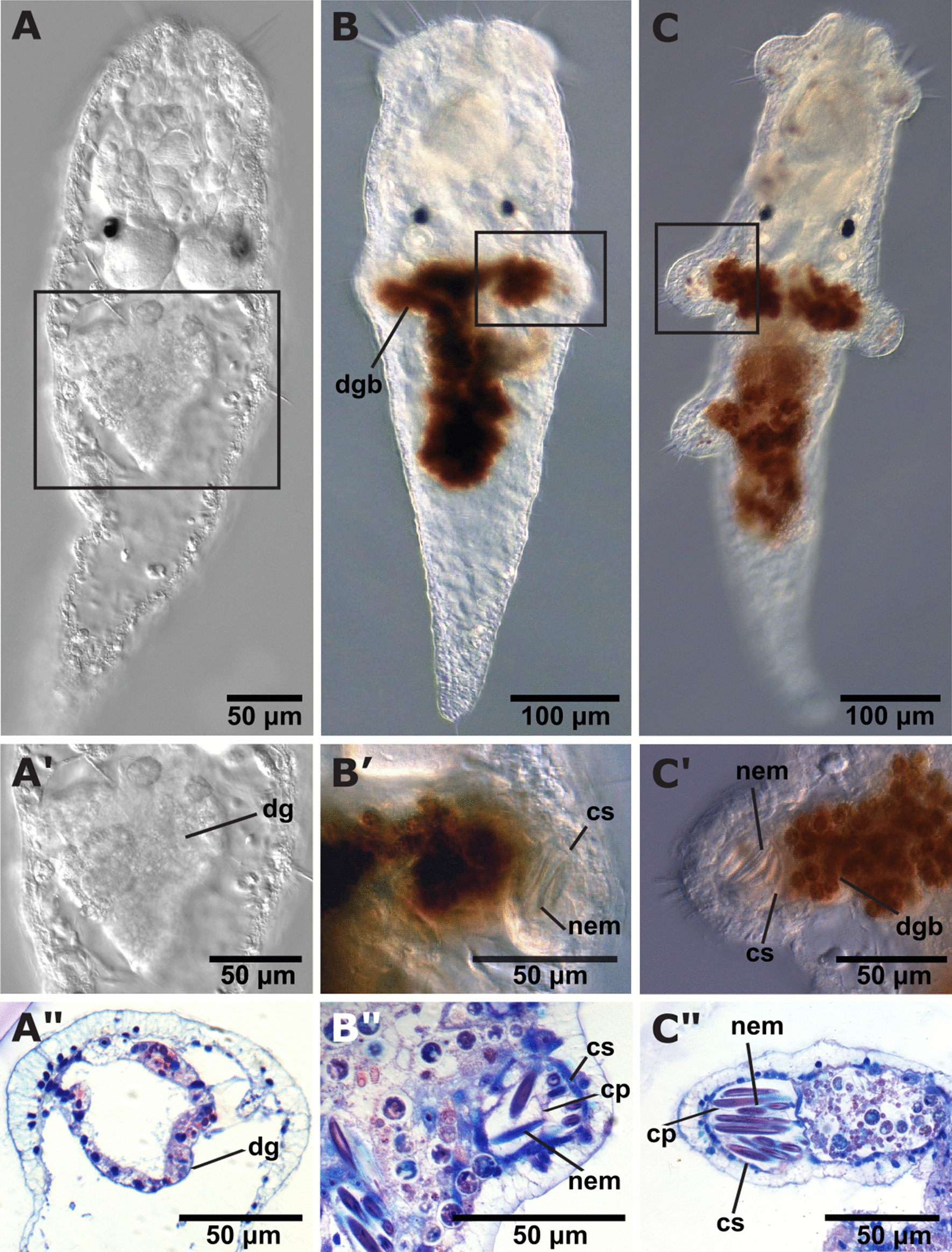
DIC and histology images of early stage *Berghia stephanieae* juveniles. These images show development of the cnidosac and beginning of nematocyst sequestration: **A** pre-feeding juvenile, **B** juvenile with cnidosacs and small cerata buds, and **C** juvenile with two rows of cerata. (**A’**–**C’**) are close up images of the digestive gland (**A’**) or cnidosac (**B’**–**C’**) in the whole animal DIC images. (**A”**–**C”**) are histological sections of animals of the same stage (SIO-BIC M18641, M18643, and M18646, respectively) showing the digestive gland (**A”**) or cnidosac (**B”**–**C”**) of those animals. Abbreviations: cp, cnidophage; cs, cnidosac; dg, digestive gland; dgb, digestive gland branch; nem, nematocyst(s)

**Fig. 4:**
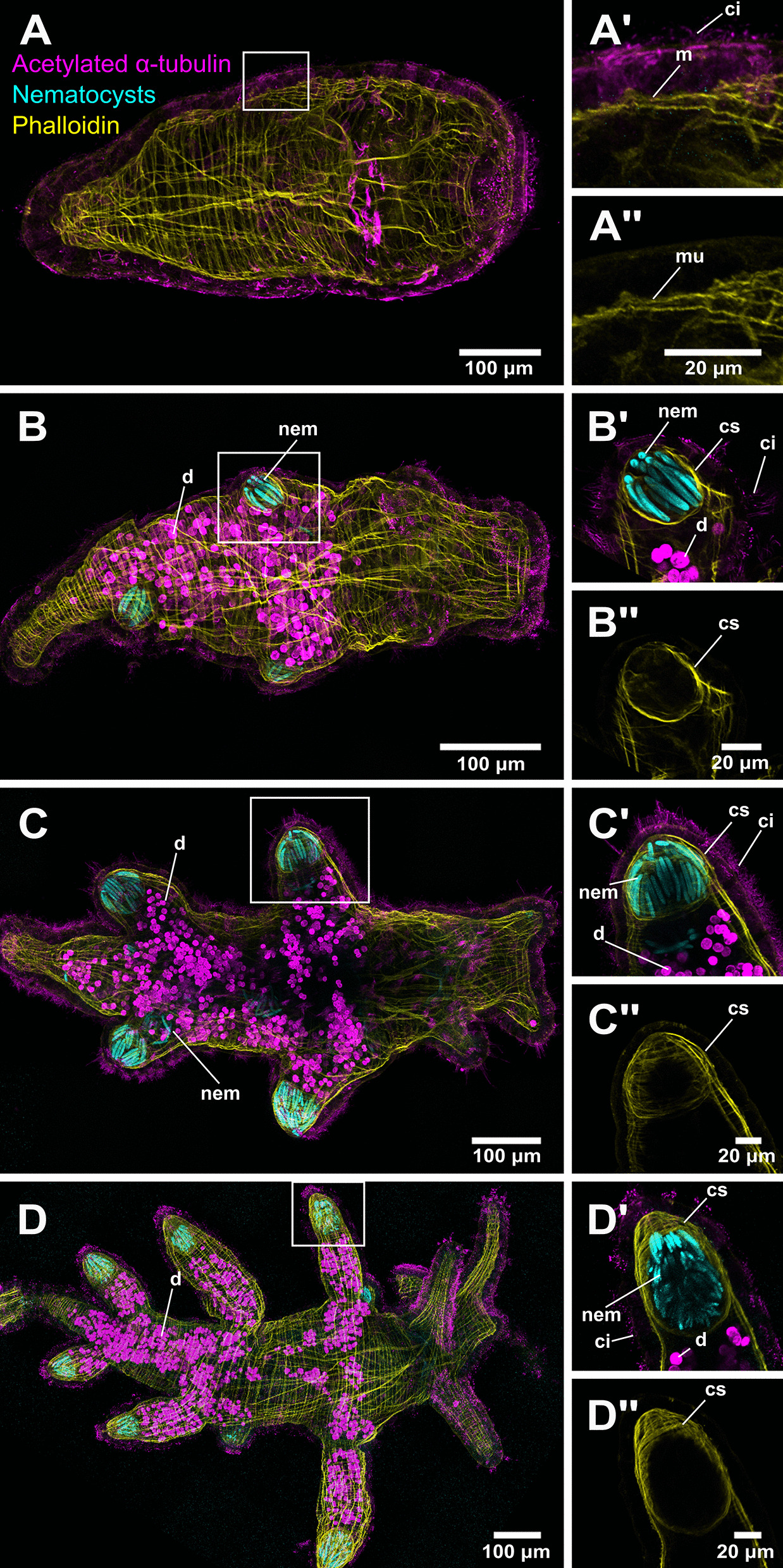
Confocal images of early stage *Berghia stephanieae* juveniles. These images show development of the cnidosac and beginning of nematocyst sequestration: **A** pre-feeding juvenile, **B** juvenile with cnidosacs and small cerata buds, **C** juvenile with two rows of cerata, and **D** juvenile with three rows of cerata. Animals were stained with acetylated alpha tubulin for cilia (magenta), DAPI for nematocysts (cyan), and Phalloidin for muscle (yellow). Some additional autofluorescence can be seen in **B**–**D** from the dinoflagellates in the gut of each animal. (**A’**–**D’** and **A”**–**D”**) are close up images of the epithelium (**A’** and **A”**) or a cnidosac (**B’**–**D’** and **B”**–**D”**) from the whole animal confocal images, and (**A”**–**D”**) only show the Phalloidin (yellow) channel to highlight the musculature of the cnidosacs. Abbreviations: ci, cilia; cs, cnidosac; d, dinoflagellates; m, muscle; nem, nematocyst(s)

Post-metamorphosis, *Berghia stephanieae* juveniles have elongate, translucent bodies with visible eyespots, statocysts, and ganglia, as well as large anterior cilia (Fig. 3A–A”). The digestive gland in these animals is compact and lacks the distinctive diverticula that define Cladobranchia as a group [36]. No cnidosacs are visible at this stage; the juveniles have not fed, and thus have a completely empty gut (Figs. 3A–A” and 4A–A’). The body wall musculature does not show any signs of cnidosac formation (Fig. 4A’–A”). Further development does not continue until, and unless, the animals begin to feed on *E. diaphana*.

Within 2–4 days post-feeding at room temperature, the digestive gland in the juveniles is filled with dinoflagellates and partially digested *Exaiptasia* tissue and nematocysts (Fig. 3B). The dinoflagellates inside the digestive gland are the most visible components of the food due to their distinctive brown coloration (when viewed with DIC optics) and bright autofluorescence (magenta in confocal images). After initial feeding, the digestive gland begins to branch away from the original compact mass, forming two digestive diverticula just behind the statocysts and sometimes a third branch more posteriorly (Fig. 3B). The cnidosacs are present at the distal end of the digestive diverticula at this stage (Fig. 3B’) with nematocysts already sequestered (Fig. 4B–B’) inside cnidophages (Figs. 3B”). At this time there is a clear extension of the body wall musculature surrounding the nascent cnidosac, inside which nematocysts are sequestered (Fig. 4B’–B”). This extension will become the cerata as the juveniles mature. Autofluorescent dinoflagellates are present in the digestive gland but are never seen inside the cnidosac.

Further development (up until the adult stage) includes the elongation and growth of the digestive diverticula, cerata, and cnidosacs (Figs. 3C–C”, 4C–C”, and 4D–D” show early juvenile stages J2–J3). As the cnidosacs grow larger, more nematocysts can be seen inside each cnidosac (Figs. 4C–C’ and D–D’) and cilia are visible at the tip of each ceras and along the sides. In some cases, nematocysts are also visible inside the digestive gland of the animal (Fig. 4C). The musculature surrounding the cnidosac becomes slightly thicker as the animals reach later stages (> 2 rows of cerata) (Fig. 4D–D”). However, the muscle layer is still not as thick as that of the cnidosacs in adult *B. stephanieae* (Fig. 1B)*.* At later stages, juveniles (Fig. 4C–C”, and D–D”) also begin adding further rows of cerata (usually symmetrically) at the posterior end of the animal as the juvenile grows longer. After the third row of cerata has formed, additional rows will be added symmetrically along the length of the animal. In these cases, both the branched digestive gland and a small cnidosac are visible.

### Feeding and nematocyst movement

**Fig. 5:**
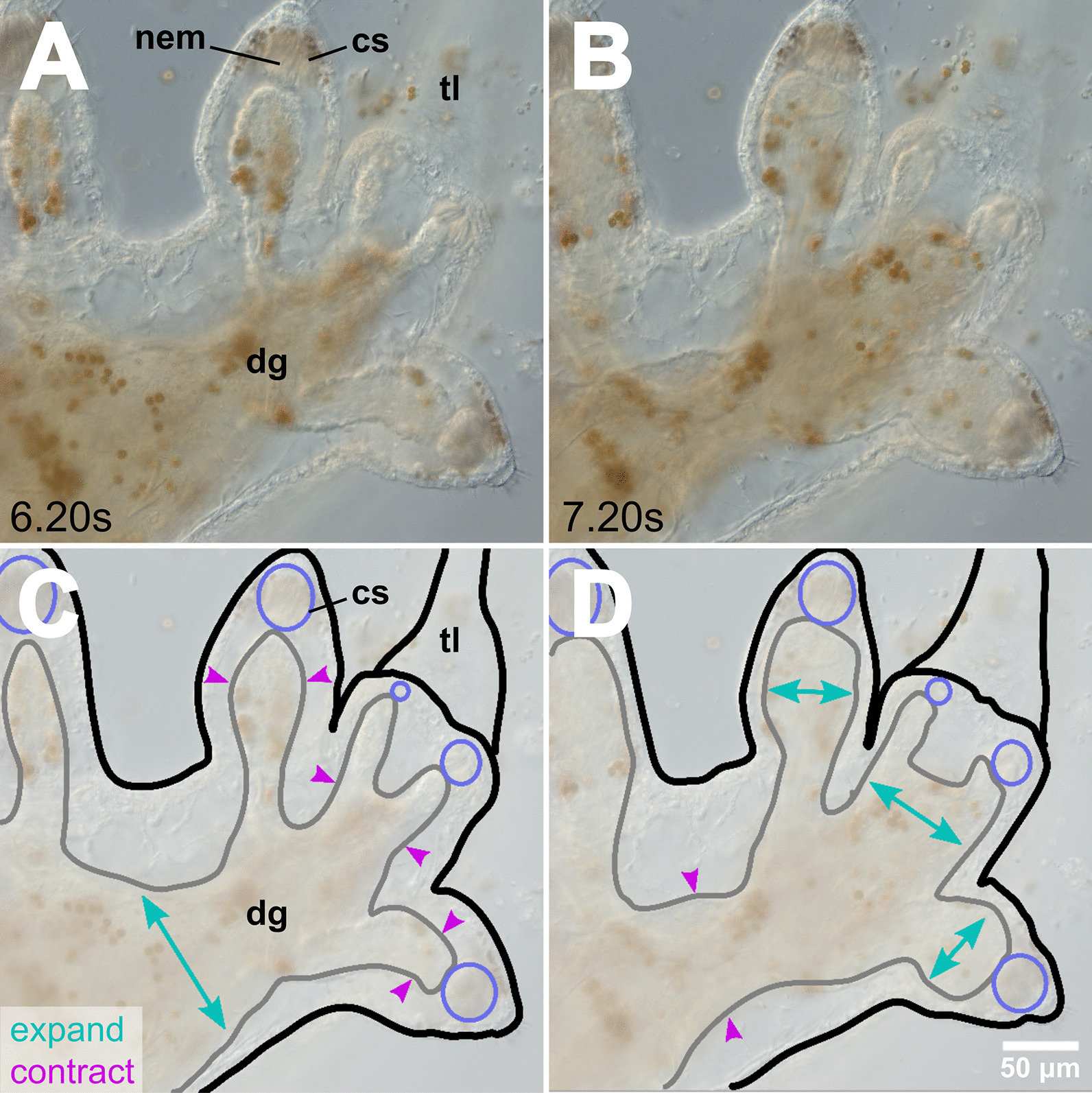
*Berghia stephanieae* juvenile digestion. Video stills (**A**, **B**) and cartoons (**C**, **D**) showing the peristaltic expansion and contraction of the digestive gland (grey outline) in a *B. stephanieae* juvenile over the course of one second (6.20–7.20 s in Additional file 2: Video 1), which occurs to move food to the distal ends of the diverticula. **A** and **C** show the expansion (teal arrows) of the primary digestive gland mass in the center of the animal, along with the contraction (pink arrows) of the diverticula. **B** and **D** show the subsequent contraction of the primary digestive gland mass in the center of the animal, along with the expansion of the diverticula. The cnidosacs (purple, **C**–**D**) do not fluctuate in size. Abbreviations: cs, cnidosac; dg, digestive gland; nem, nematocyst(s); tl, tail

*Berghia stephanieae* juveniles begin to feed on *Exaiptasia diaphana* by seemingly attaching the anterior surface of the inner lip to their prey (Additional file 2: Video 1). This is followed by a slight raising of the head and eversion of the radula, which scrapes tissue off of the surface of the *Exaiptasia*. Finally, the radula is pulled back in toward the jaws, bringing some of the *E. diaphana* tissue through the oral tube and pharynx and into the buccal cavity. The masticated food particles then move into the stomach, where they are rapidly propelled throughout the digestive gland and into the digestive diverticula through contractions of the gut (Fig. 5). As the animal continues to eat, the contractions of the gut move food particles into and out of the diverticula, until the digestive gland is completely full, or feeding is disrupted, or there is no more food to eat.

**Fig. 6:**
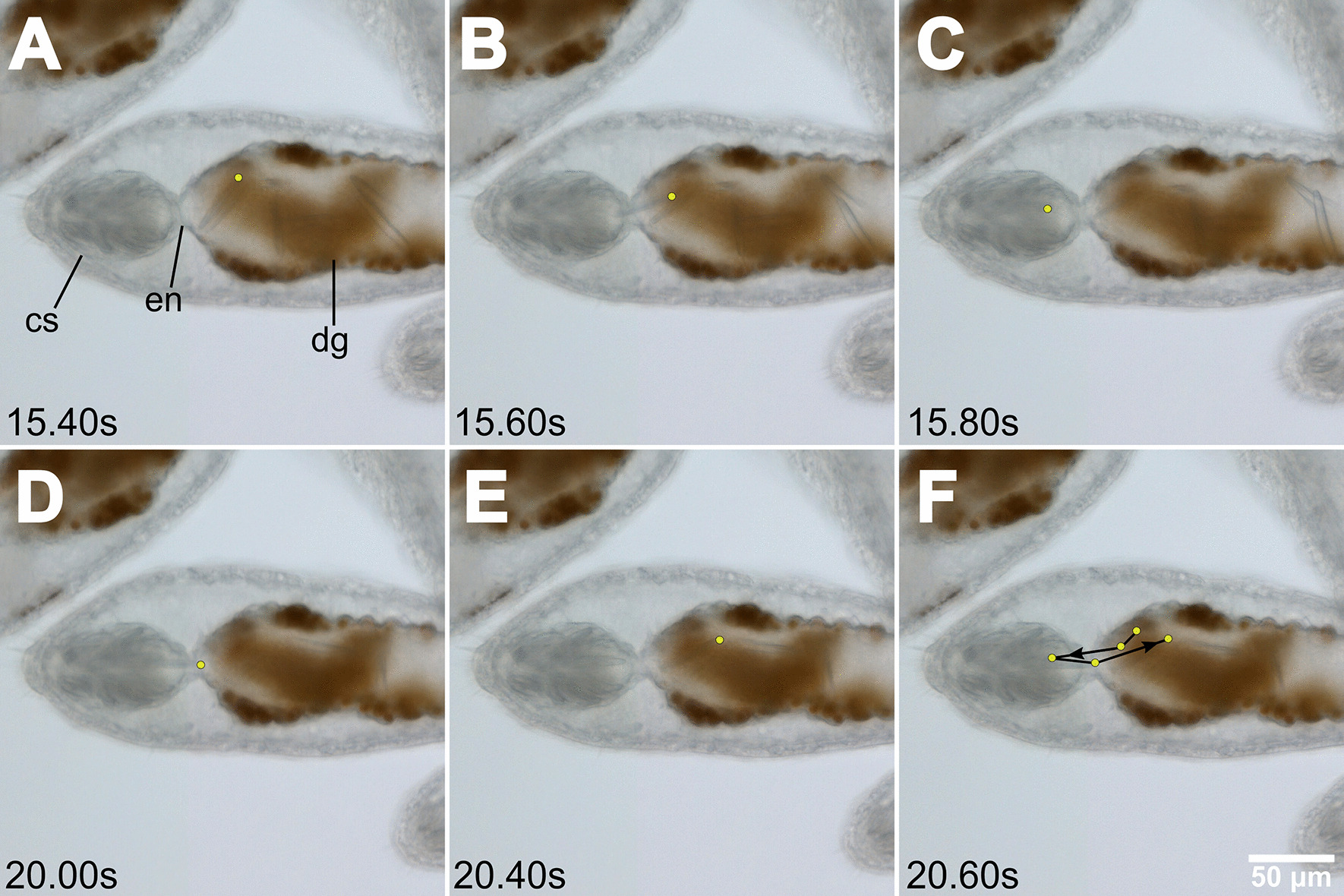
Nematocyst movement in a *Berghia stephanieae* juvenile. Video stills (Additional file 3: Video 2) showing the rapid movement of nematocysts (fractions of a second) both into and out of the cnidosac via contractions of the digestive gland. In **A**–**C** a nematocyst enters the cnidosac (yellow dots) within 0.4 s; In (**D**–**F**), the same nematocyst moves out of the cnidosac in 0.6 s; (**F**) Shows the full path of the nematocyst through time. Abbreviations: cs, cnidosac; en, entrance; dg, digestive gland

**Fig. 7:**
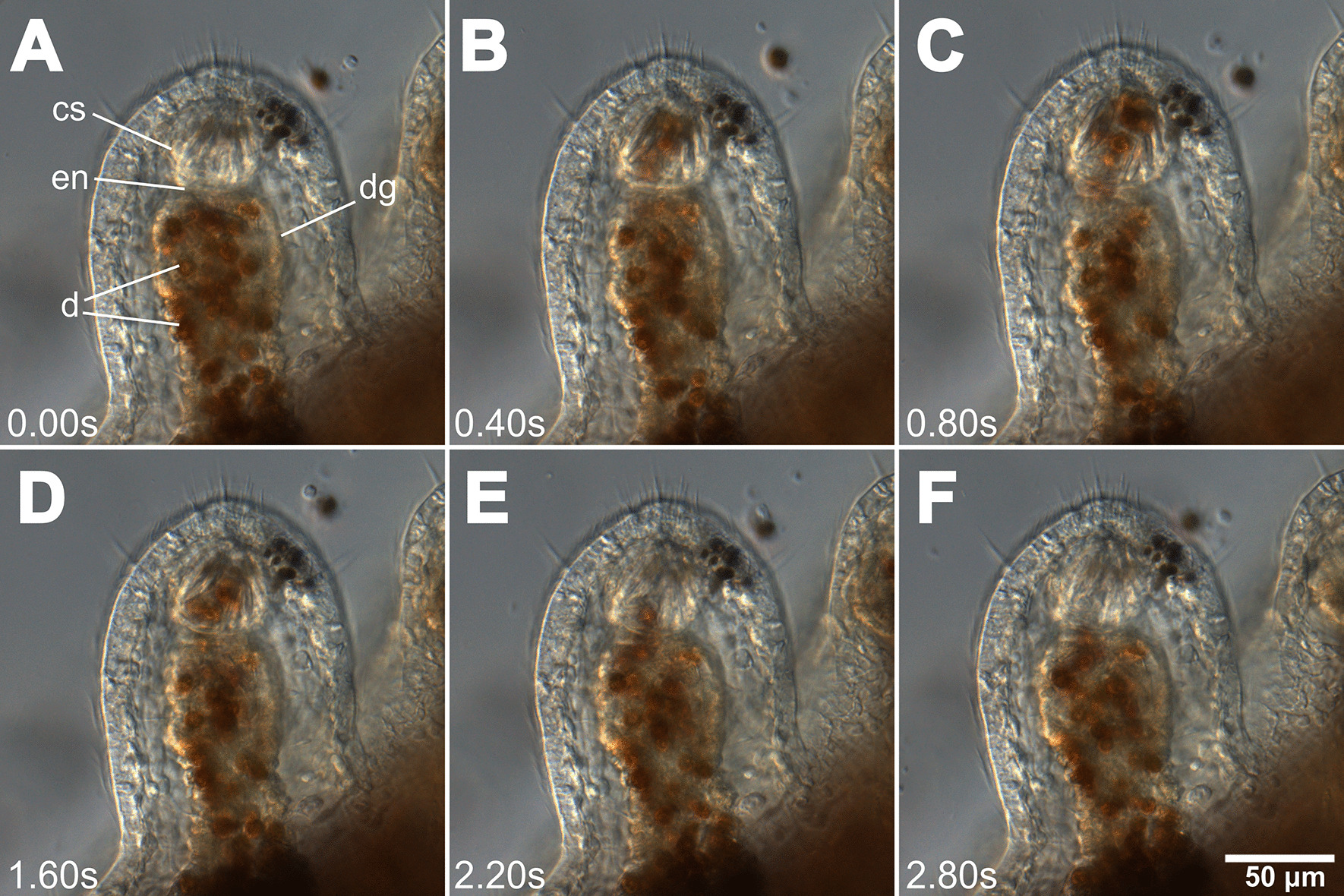
Dinoflagellate movement in a *Berghia stephanieae* juvenile. Video stills (Additional file 4: Video 3) showing the rapid movement (fractions of a second) of dinoflagellates (brown circles) both into and out of the cnidosac via contractions of the digestive gland. In (**A**–**C**) dinoflagellates move into the cnidosac within 0.8 s; In (**D**–**F**), the same dinoflagellates move out of the cnidosac in 1.2 s. Abbreviations: cs, cnidosac; en, entrance; d, dinoflagellates, dg, digestive gland

As this process continues, the channel between the digestive gland and the cnidosac opens and closes with the contractions of the gut (Fig. 6). These contractions provide the opportunity for nematocysts to move into the cnidosac, often at a rapid pace (~ 0.4 s in Fig. 6A–C). However, the large contractions and expansions that occur in the stomach and digestive gland can also act to move nematocysts out of the cnidosac (Fig. 6D–F) at an equally rapid rate. This rapid movement is also true for other food particles inside the digestive gland, including intact dinoflagellates (Fig. 7; Additional file 3: Video 2). Furthermore, both nematocysts and dinoflagellates can move into the cnidosac (Figs. 6, 7).

## Discussion

**Fig. 8:**
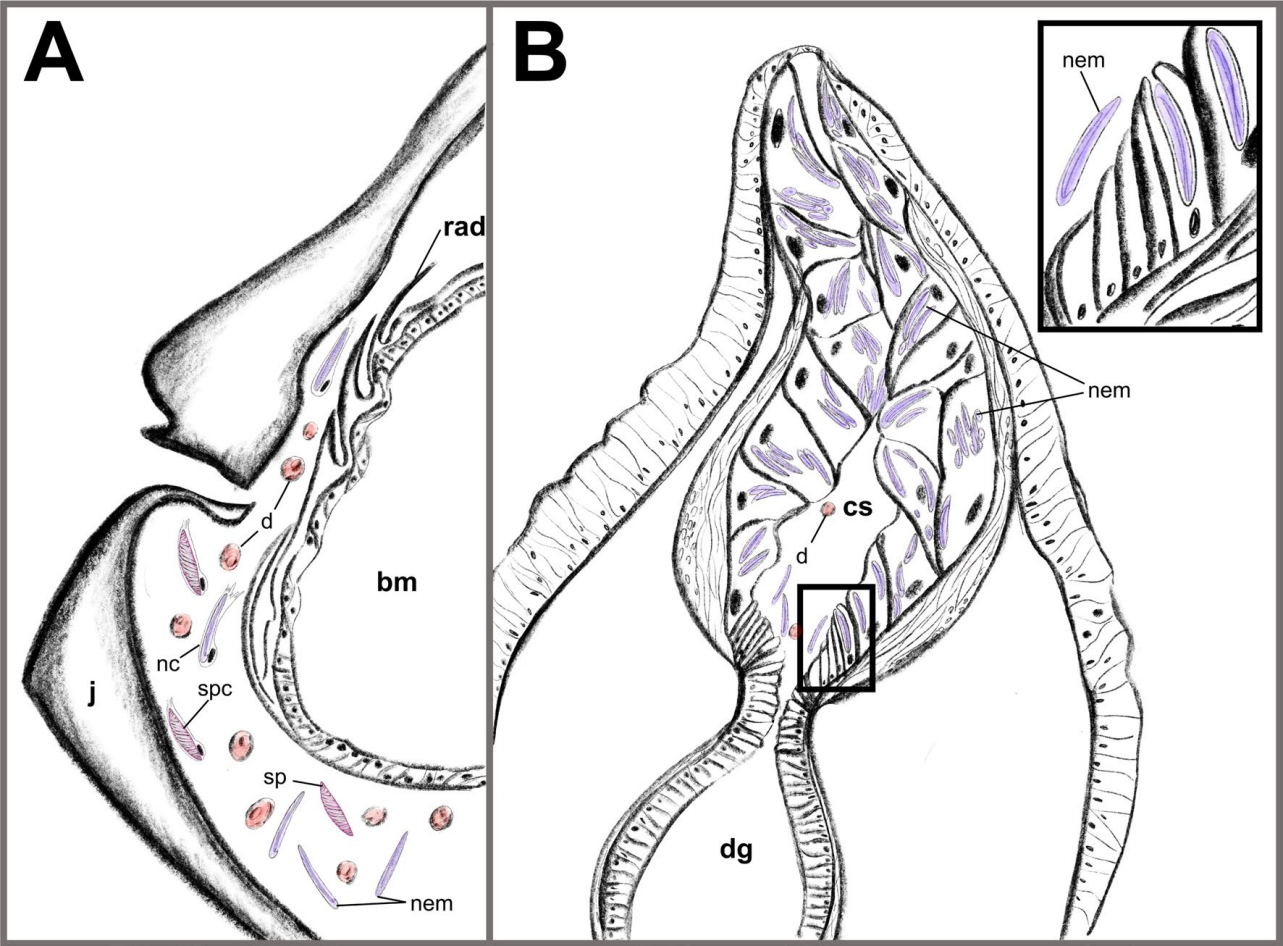
Schematic highlighting our hypothesis for nematocyst sequestration in *Berghia stephanieae* adults. **A** Early in the digestive system (e.g., oral tube, pharynx, or stomach), nematocysts (the organelle) are stripped of their nematocyte (the cell) and then enter the digestive gland. **B** The distal end of a *B. stephanieae* ceras showing the cnidophages in the proximal end of the cnidosac (inset) identify and phagocytose nematocysts over other structures after they enter the cnidosac. Abbreviations: bm, buccal mass; cs, cnidosac, d, dinoflagellates, dg, digestive gland; j, jaws; nc, nematocyte(s); nem, nematocyst(s); rad, radula; sp, spirocyst(s); spc, spirocyte(s)

Nematocyst sequestration represents an excellent opportunity to investigate how ubiquitous cellular mechanisms like phagocytosis can be modified to selectively internalize and store extrinsic structures. However, laboratory models for nematocyst sequestration have long remained elusive, largely due to difficulties in culturing nudibranch species in the laboratory, including low levels of settlement and metamorphic success in the absence of specific environmental cues [37–39] and limitations on culturing the prey for a given species [40]. With *Berghia stephanieae,* these challenges have been overcome due to its lecithotrophic development [41, 42] and dietary preference for *Exaiptasia diaphana* [40, 42], a cnidarian species that is simple to culture under laboratory conditions [43]. Here, we show that the nudibranch species *B. stephanieae* is an excellent model for nematocyst sequestration, in part due to the ease of access to early developmental stages where juveniles are more transparent and manipulable. These tools have allowed us to address the “when” and “where” of selectivity for nematocysts in *B. stephanieae*. Our results clearly indicate that in *B. stephanieae*, nematocyst sequestration begins shortly after feeding and prior to ceras formation, and nematocyst selectivity occurs inside the cnidosac, likely within the cnidophage cells. We discuss the importance of these findings below, and propose a hypothesis for nematocyst sequestration in *B. stephanieae* where the nematocyte (the cell) membrane is removed during early digestion (Fig. 8A), and only nematocysts (the organelles) are targeted and phagocytosed by cnidophages (Fig. 8B).

### The when—cnidosac development

The morphology of the adult cnidosac in *Berghia stephanieae* (Fig. 1A, B) is broadly consistent with previous work in this species, and in the family Aeolidiidae [25, 44]. This morphology includes a short, simple entrance into the cnidosac from the digestive gland, a thick (multi-layered) musculature surrounding the cnidosac, a putative proliferation zone of cnidophage cells at the proximal end of the cnidosac [25], multiple nematocysts housed within cnidophage cells along the lining of the cnidosac. One double membrane is present around each individual nematocyst in the cnidosac (Fig. 1E), supporting the longtime assertion that nematocysts (the organelles) are sequestered, rather full and intact nematocytes (the cells). Nematocysts are likely divested of their nematocyte earlier in the digestive system of *B. stephanieae*, possibly in the pharynx (Fig. 8A). If the whole cell were sequestered, we would expect to see two double membranes (the nudibranch membrane and the cnidarian membrane). An exit was identified, but, *B. stephanieae* does not have a clear epithelial lining inside the cnidosac toward the tip (*i.e.,* a cnidopore), which is a structure commonly found in members of Aeolidiidae, including *Aeolidia papillosa* [44], *Anteaeolidiella chromosoma*, and *Cerberilla amboinensis* [25]. The cnidopore has been hypothesized to be a special adaptation for releasing the exceptionally long and narrow nematocysts sequestered from anemones [25]. The lack of this structure in *B. stephanieae* suggests that this is not a necessary feature for managing such nematocysts.

Sequestered nematocysts appear within fully-formed cnidosacs within 2–4 days after feeding. Cnidosacs are defined as a “muscular prolongation of the digestive gland located in the apex of each ceras” [25]. In *B. stephanieae* juveniles, digestive diverticula (branches) and cnidosacs develop before the beginning of cerata formation (Figs. 3B–B”, 4B–B”). These early stage cnidosacs, prior to ceras formation, already have a clear musculature (Fig. 4B”, C”). Furthermore, nematocysts are also clearly bound inside what appear to be cnidophages (Fig. 3B’–B”), indicating that, if not fully mature, these cnidosac structures are at least fully functional. These results differ from regeneration work in other nudibranch species, including *Hermissenda crassicornis* [45] and *Pteraeolidia semperi* [46]. In both species, experiments following the regeneration of cerata after autotomy (or removal) suggested that cerata and the digestive gland form first, followed by the cnidosac [45, 46]. In *Pteraeolidia semperi*, cnidosacs were documented regenerating from a cell aggregation at the tip of the regenerating digestive tract [46]. A prior study on *B. stephanieae*, focused on neuromuscular development, also incorrectly identified the timing of cnidosac development after ceras formation, based on the presence of filled cnidosacs under light microscopy [41]. These inconsistencies may be due to differences among species [46], differences between regeneration and development [45, 46], or lower resolution imaging techniques [41, 45, 46].

Our results show that nematocyst sequestration in *B. stephanieae* begins much earlier than previously thought. This fact will be useful for studies in *B. stephanieae* in the long term, because the cerata and cnidosacs at these early juvenile stages are more accessible visually due to less external pigmentation relative to later stages [41]. Similarly, our ability to detect nematocyst sequestration this early in juvenile development means that in the future we could score gene knock-out phenotypes (e.g. generated by CRISPR/Cas9 genome editing) at juvenile stages. Clarification of the timing of nematocyst sequestration may also allow for the use of transient knock-down methods in *B. stephanieae*, such as morpholinos or RNA interference [47]. *B. stephanieae* juveniles therefore promise to be an excellent tool for investigating the cell and molecular processes involved in nematocyst sequestration.

### The where—nematocyst selectivity

Our results show that *Berghia stephanieae* selectively sequesters nematocysts (over spirocysts or dinoflagellates) inside the cnidosac (Fig. 2). Most studies on nematocyst sequestration in nudibranchs have assumed, but not demonstrated, that only nematocysts are sequestered, or have not clarified the type of cnidocysts found within nudibranch cnidosacs [25]. Three types of cnidocysts are produced in the phylum Cnidaria: nematocysts, spirocysts, and ptychocysts. Nematocysts are the most widespread across the phylum, with spirocysts and ptychocysts only known from Hexacorallia (and ptychocysts only from the hexacorallian order Ceriantharia) [48]. The lack of clarification regarding which cnidocysts are sequestered may be due to the fact that few nematocyst-sequestering nudibranchs feed on cnidarians known to possess ptychocysts or spirocysts (*i.e.,* Hexacorallia) [49]. However, nudibranchs in Aeolidiidae feed on anemones (including *Exaiptasia diaphana*), which possess high concentrations of spirocysts in their tentacles for prey capture (*e.g.,* Fig. 2C) [50, 51]. We know of only one study that suggested the presence of spirocysts inside the cnidosac, which were identified in specimens of an undescribed species of *Spurilla* from Argentina [52]. However, no images of these structures were provided, so independent confirmation is impossible. The selectivity for nematocysts we find in *B. stephanieae*, however, may be due to the more defensive (and offensive) function of nematocysts in cnidarians. Nematocysts deliver venom as a means of defense and offense [48], unlike spirocysts [53] and most likely ptychocysts [54]. Therefore, selectivity of nematocysts over less potent structures like spirocysts provides support for a defensive function of nematocyst sequestration in nudibranchs, which is a long-standing debate (see references in [5]).

In addition to differentiating cnidocyst types (spirocysts versus nematocysts), we further identified the types of nematocysts sequestered in the *B. stephanieae* cnidosac, including basitrichous isorhizas, large microbasic p-mastigophores, and microbasic p-amastigophores of varying sizes (Additional file 1: Figures S2 and S3). Based on previous work in *Exaiptasia diaphana* (*Exaiptasia pallida* in [34]) and the size and type of the nematocysts found in *B. stephanieae* (Fig. 1C, D; Additional file 1: Figures S2 and S3), we have evidence of the sequestration of nematocysts from multiple *E. diaphana* tissues (including the acontia and tentacles). In some nudibranch species, researchers have found that certain nematocyst types are preferentially sequestered in the cnidosacs [20, 21, 55, 56]. For example, larger, more penetrant nematocyst types [57, 58] such as mastigophores and long isorhizas, appear to be preferred in some species. We found no direct evidence suggesting *B. stephanieae* select for particular types of nematocysts, though large microbasic p-mastigophores were prevalent in the cnidosacs. Future studies performing more quantification of nematocyst types compared to their distribution across prey tissues will be necessary to test this hypothesis further in *B. stephanieae*.

In *B. stephanieae*, our results support the hypothesis that selectivity for nematocyst occurs within the cnidosacs (Figs. 6, 7), most likely within the cnidophages [59]. This support stems from the fact that both nematocysts (Fig. 6) and dinoflagellates (Fig. 7) can enter the cnidosac during digestion, without first being internalized within digestive cells. Other hypotheses propose that nematocysts are selected for by modified digestive cells inside the digestive gland, and move along the digestive tract to the cnidosac [30], or that the entrance of the cnidosac (which is surrounded by a muscular sphincter [30]) is a barrier to all structures except for nematocysts. Given our evidence for selectivity occurring inside the cnidosac (Figs. 6, 7), we now know to focus on cnidophages when investigating the molecular mechanisms of nematocyst sequestration (Fig. 8B). In cnidarian-dinoflagellate endosymbiosis, recognition and phagocytosis appear to be mediated by microbe-associated molecular pattern (MAMP)-pattern recognition receptors (PRR) that target a variety of molecular patterns on the cell surface of potential symbionts [3]. Similar interactions may also be used to identify plastids in Sacoglossa sea slugs that sequester functional choloroplasts [60]. But in nudibranchs, nematocysts that are selected for and phatocytosed by cnidophages have been stripped of their nematocyte [4, 30]. This raises questions as to whether cnidophages can also use MAMP-PRR interactions to select for nematocysts over other tissues and structures from their prey (*e.g.,* spirocysts or dinoflagellates). *B. stephanieae* would be an excellent system for investigating how phagocytosis has been modified to select for structures that have been stripped of their original cell.

## Conclusions

Nematocyst sequestration is an excellent system for studying direct ecological interactions of predators and prey at the cell and molecular levels. Here, we show that the nudibranch *Berghia stephanieae* is an incredibly useful experimental research organism for studying nematocyst sequestration, due in part to the success of lab cultures for this species. Furthermore, our results show that the developmental and cell biology of nematocyst sequestration can be thoroughly investigated in *B. stephanieae.* We show that nematocyst sequestration begins shortly after feeding and prior to ceras formation in this species, which will be useful for future studies aimed at visualizing or disrupting the process of nematocyst sequestration. Finally, we show that selectivity for nematocysts (over other structures like spirocysts) occurs inside the cnidosac in *B. stephanieae*, likely in the cnidophage cells. With this information, we can begin to investigate whether the cnidophages use immune processes like MAMP-PRR recognition to identify nematocysts. If so, this would allow us to directly investigate how common and ubiquitous cellular mechanisms (like phagocytosis) can be modified to selectively internalize and store extrinsic structures based on external cues.

## Methods

### Animal maintenance and preparation

Adults of *Berghia stephanieae* were maintained in continuous culture in the Lyons Lab at the Scripps Institution of Oceanography, broadly following techniques laid out by Carroll and Kempf [42]. Animals were kept at a salinity of 1.024 sg in artificial seawater (Instant Ocean, Spectrum Brands, Blacksburg, VA) at room temperature (~ 20 °C) in finger bowls. We prepared ASW with Instant Ocean (Reef Crystals Reef Salt) to a salinity of 33 ppt and a specific gravity of 1.024. *B. stephanieae* animals were fed *Exaiptasia diaphana* three days per week (Monday, Wednesday, Friday). The amount of food varied based on animal size: For example, adults were fed whole *Exaiptasia* (~ 1 cm pedal disc diameter) per 6 animals, and just metamorphosed juveniles were fed 3–5 *Exaiptasia* tentacles per hundreds of animals. *E. diaphana* were purchased from Carolina Biological Supply, Burlington, NC.

Prior to fixation for histology or antibody staining, we relaxed juveniles of *B. stephanieae* in a 1 part 7.3% MgCl_2_ to 2 parts Artificial Sea Water (ASW) solution for ~ 20 min. For live imaging, we did not perform a relaxation step. We initially staged juveniles based on previous descriptions of juvenile development [41], and selected animals between the early juvenile and juvenile stages, as these are the stages where the animals begin feeding and cerata emerge.

### Histology

Post-relaxation, we fixed juveniles and adult cerata using 4% Paraformaldehyde (PFA, diluted in ASW from 16% ampules) overnight at 4 °C, then rinsed twice in diH_2_O (30 s each). We performed a post-fixation stain using Ponceau S (0.1% Ponceau S and 1.0% glacial acetic acid) for 1.5 h, followed by a diH_2_O rinse. We then immediately ran the tissues through a dehydration series: (1) 50% ethanol (EtOH) for 15 min, (2) 60% EtOH for 15 min, and (3) 70% EtOH for 15 min. We stored tissues at 70% EtOH prior to embedding. In preparation for embedding, we subjected tissues to a further dehydration series: (1) 80% EtOH for 15 min, (2) 95% EtOH for 15 min, (3) 100% EtOH for 15 min (×2). We embedded samples with Spurr's Low Viscosity Embedding Media Kit (Electron Microscopy Sciences #14,300), following the standard “Firm” formulation provided by the manufacturer, and cured at 60 °C overnight.

We sectioned the plastic blocks with a microtome and glass knives, to a thickness of 3 µm. We then stained sections with Azure A for ~ 30 s. We rinsed slides with diH_2_O for 30 s before placing them on a hot plate to dry. We then mounted the sections in Permount (Fisher Scientific, SP15-100) for long-term storage and imaging. We took section images on a Zeiss AxioM2 fluorescence microscope with an attached digital camera (Axiocam 506 color). We combined image stacks in Helicon Focus (version 7.7.5), adjusted images for brightness and contrast using Fiji (version 2.3.0, [61]), and prepared image plates using the FigureJ plugin [62]. We histologically examined at least two cnidosacs from five separate adult *B. stephanieae* (and ~ 20 cnidosacs in one animal), and at least three juveniles from each of the four stages.

### Transmission electron microscopy

Post-relaxation, samples were sent to the Electron Microscopy Core Facility at the University of California, San Diego. They fixed adult cerata with 2% paraformaldehyde and 2.5% of glutaraldehyde in 0.15 M Sodium Cacodylate buffer (SC buffer, pH 7.4) for at least 24 h. They stained tissues in 1% osmium in 0.15 M sodium cacodylate for 1–2 h on ice, followed by a 5 × 10 min wash in 0.15 M SC buffer and rinse in ddH2O on ice. They then incubated the samples in 2% of uric acid for 1 to 2 h at 4 °C, followed by a dehydration series in ethanol (50%, 70%, 90%, 2 × 100%), with 10 min on ice for each step. They then placed the samples in dry acetone for 15 min at room temperature (RT), followed by 50:50 (ethanol:Durcupan) for 1 h or longer at RT and 100% Durcupan overnight. The following day, they moved the samples into fresh 100% Durcupan for half a day at RT and embedded tissues in Durcupan in 60 °C oven for 36 to 48 h. They collected ultrathin Sects. (60 nm), which they cut on Leica microtome with Diamond knife, followed by post staining with both uranyl acetate and lead. We captured images on a JEOL 1400 plus TEM at 80 kV with Gatan 4kx4k camera at the facility. We prepared image plates using the FigureJ plugin [62].

### Fluorescence imaging

Post-relaxation, we fixed juveniles using 4% Paraformaldehyde (PFA) overnight at 4 °C. We rinsed samples with PBT (0.1% Triton-X in 1X Phosphate Buffered Saline (PBS)) for 10 min (× 3) and incubated either overnight at 4 °C or for 2 h at room temperature (RT) in blocking solution (500 µL Bovine Serum Albumin, 400 µL Normal Donkey Serum, 9.1 mL 1X PBS with 0.1% Tween). We then placed samples in acetylated alpha-tubulin primary antibody (Anti-mouse; Sigma-Aldrich T6793) (diluted in blocking solution) overnight at 4 °C, rinsed three times in PBT, then incubated in secondary antibody (Anti-mouse, Alexa Fluor 594) overnight at 4 °C (incubated in blocking solution). Finally, we stained tissues with a high concentration of DAPI (143 µM; ThermoFisher Scientific #D1306) to stain nematocysts [63], and Alexa Fluor 488 Phalloidin (1:500; ThermoFisher Scientific #A12379) prior to 1X PBS rinses (× 3) and mounting in a solution containing 80% glycerol and 20% 1X PBS. We performed imaging with a Leica Sp8 inverted confocal microscope equipped with a 20X objective (0.70 DRY) and a resonant scanner. Nematocysts were detected by exciting DAPI with a 448 nm laser (rather than the usual 405 nm) and an emission filter collecting light at a peak of 481 nm (CFP range). This method allows detection of the high intensity DAPI signal in the nematocysts without detecting the lower intensity nuclear signal. We performed deconvolution of the datasets with the Leica Lightning deconvolution software, and visualized images using Fiji (version 2.3.0, [61]), and prepared image plates using the FigureJ plugin [62].

### Live Imaging

For live imaging, we placed juveniles on uncoated glass slides with small fragments of *Exaiptasia diaphana* tentacles. We then covered the animals with a coverslip held up by clay feet but refrained from applying pressure until after the *B. stephanieae* juveniles began to eat. Once they started eating the *Exaiptasia* tentacles, we pressed down on the corners of the coverslip until the digestive gland and cnidosacs of the animals were easily visible under the microscope. We took live videos of these juveniles with a Zeiss AxioM2 microscope with a 20X objective and a digital camera (Axiocam 506 color).

## Supplementary Information

**Additional file 1**. Supplementary Figures S1–3.

**Additional file 2**. Video showing the peristaltic expansion and contraction of the digestive gland in a *B. stephanieae* juvenile over the course of one second (6.20-7.20s), which occurs to move food to the distal ends of the diverticula. Stills presented in Fig. 5.

**Additional file 3**. Video showing the rapid movement of nematocysts (fractions of a second) both into and out of the cnidosac via contractions of the digestive gland in a* B. stephanieae *juvenile. Stills presented in Fig. 6.

**Additional file 4**. Video showing the rapid movement (fractions of a second) of dinoflagellates (brown circles) both into and out of the cnidosac via contractions of the digestive gland in a *B. stephanieae *juvenile. Stills presented in Fig. 7.

## Data Availability

Most data generated or analysed during this study are included in this published article and its additional files. Histological slides and vouchers are available from the Scripps Institution of Oceanography Benthic Invertebrate Collection (SIO-BIC) for both *Berghia stephanieae* (slides, M18637-M18646; vouchers, M18660-M18661) and *Exaiptasia diaphana* (Co3615-Co3617).

## References

[1] Pelletreau KN, Bhattacharya D, Price DC, Worful JM, Moustafa A, Rumpho ME (2011). Sea slug kleptoplasty and plastid maintenance in a metazoan. Plant Physiol.

[2] Jesus B, Jauffrais T, Trampe ECL, Goessling JW, Lekieffre C, Meibom A (2022). Kleptoplast distribution, photosynthetic efficiency and sequestration mechanisms in intertidal benthic foraminifera. ISME J.

[3] Davy SK, Allemand D, Weis VM (2012). Cell biology of cnidarian-dinoflagellate symbiosis. Microbiol Mol Biol Rev.

[4] Greenwood PG (2009). Acquisition and use of nematocysts by cnidarian predators. Toxicon.

[5] Goodheart JA, Bely AE (2017). Sequestration of nematocysts by divergent cnidarian predators: mechanism, function, and evolution. Invertebr Biol.

[6] Rosset SL, Oakley CA, Ferrier-Pagès C, Suggett DJ, Weis VM, Davy SK (2021). The molecular language of the cnidarian-dinoflagellate symbiosis. Trends Microbiol.

[7] Moné Y, Monnin D, Kremer N (2014). The oxidative environment: a mediator of interspecies communication that drives symbiosis evolution. Proc Biol Sci.

[8] van Oppen MJH, Oliver JK, Putnam HM, Gates RD (2015). Building coral reef resilience through assisted evolution. Proc Natl Acad Sci USA.

[9] Silverstein RN, Correa AMS, Baker AC (2012). Specificity is rarely absolute in coral-algal symbiosis: implications for coral response to climate change. Proc Biol Sci.

[10] Gabay Y, Parkinson JE, Wilkinson SP, Weis VM, Davy SK (2019). Inter-partner specificity limits the acquisition of thermotolerant symbionts in a model cnidarian-dinoflagellate symbiosis. ISME J.

[11] Jacobovitz MR, Rupp S, Voss PA, Maegele I, Gornik SG, Guse A (2021). Dinoflagellate symbionts escape vomocytosis by host cell immune suppression. Nat Microbiol.

[12] Carré D, Carré C (1980). On triggering and control of cnidocyst discharge. Mar Behav Physiol.

[13] Greenwood PG, Mariscal RN (1984). Immature nematocyst incorporation by the aeolid nudibranch *Spurilla neapolitana*. Mar Biol.

[14] Yarnall JL. The feeding behavior and functional anatomy of the gut in the eolid nudibranchs *Hermissenda crassicornis* (Eschscholtz, 1831) and *Aeolidia papillosa* (Linnaeus, 1761) [PhD]. Stanford University; 1972. Available from: https://searchworks.stanford.edu/view/2191039

[15] Tay MZ, Wiehe K, Pollara J (2019). Antibody-dependent cellular phagocytosis in antiviral immune responses. Front Immunol.

[16] Uribe-Querol E, Rosales C (2020). Phagocytosis: our current understanding of a universal biological process. Front Immunol.

[17] Savitzky AH, Mori A, Hutchinson DA, Saporito RA, Burghardt GM, Lillywhite HB (2012). Sequestered defensive toxins in tetrapod vertebrates: principles, patterns, and prospects for future studies. Chemoecology.

[18] Petschenka G, Agrawal AA (2016). How herbivores coopt plant defenses: natural selection, specialization, and sequestration. Curr Opin Insect Sci.

[19] Cimino G, Ghiselin MT (1999). Chemical defense and evolutionary trends in biosynthetic capacity among dorid nudibranchs (Mollusca: Gastropoda: Opisthobranchia). Chemoecology.

[20] Edmunds M (1966). Protective mechanisms in the Eolidacea (Mollusca Nudibranchia). J Linn Soc London, Zool.

[21] Frick K (2003). Response in nematocyst uptake by the nudibranch *Flabellina verrucosa* to the presence of various predators in the Southern Gulf of Maine. Biol Bull.

[22] Obermann D, Bickmeyer U, Wägele H (2012). Incorporated nematocysts in *Aeolidiella stephanieae* (Gastropoda, Opisthobranchia, Aeolidoidea) mature by acidification shown by the pH sensitive fluorescing alkaloid Ageladine A. Toxicon.

[23] Krohne G. Organelle survival in a foreign organism: *Hydra* nematocysts in the flatworm *Microstomum lineare*. Eur J Cell Biol. Urban & Fischer; 2018;97:289–99. 10.1016/j.ejcb.2018.04.002 29661512

[24] Krohne G (2020). *Hydra* nematocysts in the flatworm *Microstomum lineare*: in search for alterations preceding their disappearance from the new host. Cell Tissue Res.

[25] Goodheart JA, Bleidißel S, Schillo D, Strong EE, Ayres DL, Preisfeld A (2018). Comparative morphology and evolution of the cnidosac in Cladobranchia (Gastropoda: Heterobranchia: Nudibranchia). Front Zool.

[26] Valdés A (2005). A new species of *Aeolidiella* Bergh, 1867 (Mollusca: Nudibranchia: Aeolidiidae) from the Florida Keys, USA. Veliger.

[27] Carmona L, Pola M, Gosliner TM, Cervera JL (2014). The Atlantic-Mediterranean genus *Berghia* Trinchese, 1877 (Nudibranchia: Aeolidiidae): taxonomic review and phylogenetic analysis. J Molluscan Stud Oxford Academic.

[28] Dungan AM, Hartman LM, Tortorelli G, Belderok R, Lamb AM, Pisan L (2020). *Exaiptasia diaphana* from the great barrier reef: a valuable resource for coral symbiosis research. Symbiosis.

[29] Martin R (2003). Management of nematocysts in the alimentary tract and in cnidosacs of the aeolid nudibranch gastropod *Cratena peregrina*. Mar Biol.

[30] Martin R, Heß M, Schrödl M, Tomaschko K-H (2009). Cnidosac morphology in dendronotacean and aeolidacean nudibranch molluscs: from expulsion of nematocysts to use in defense?. Mar Biol.

[31] Marin A. Chemical or nematocyst-based defence in the nudibranch *Cratena peregrina*? - a reply to B.K. Penney. J Molluscan Stud. 2009;75:201–2.

[32] Penney BK. A comment on F. Aguado & A. Marin: “Warning coloration associated with nematocyst-based defences in aeolidioidean nudibranchs.” J Molluscan Stud. 2009;75:199–200.

[33] Aguado F, Marin A (2007). Warning coloration associated with nematocyst-based defences in aeolidiodean nudibranchs. J Molluscan Stud.

[34] Grajales A, Rodríguez E (2014). Morphological revision of the genus *Aiptasia* and the family Aiptasiidae (Cnidaria, Actiniaria, Metridioidea). Zootaxa.

[35] Östman C (2000). A guideline to nematocyst nomenclature and classification, and some notes on the systematic value of nematocysts. Sci Mar.

[36] Wägele H, Willan RC (2000). Phylogeny of the Nudibranchia. Zool J Linn Soc.

[37] Dionísio G, Rosa R, Leal MC, Cruz S, Brandão C, Calado G (2013). Beauties and beasts: a portrait of sea slugs aquaculture. Aquaculture.

[38] Hadfield MG, Pennington JT (1990). Nature of the metamorphic signal and its internal transduction in Larvae of the Nudibranch *Phestilla Sibogae*. Bull Mar Sci.

[39] Schlesinger A, Goldshmid R, Hadfield MG, Kramarsky-Winter E, Loya Y (2009). Laboratory culture of the aeolid nudibranch *Spurilla neapolitana* (Mollusca, Opisthobranchia): life history aspects. Mar Biol.

[40] Monteiro EA, Güth AZ, Banha TNS, Sumida PYG, Mies M (2020). Implications of feeding frequency, prey size and condition, and intraspecific competition for the commercial aquaculture of the nudibranch *Berghia stephanieae*. J World Aquac Soc.

[41] Kristof A, Klussmann-Kolb A. Neuromuscular development of *Aeolidiella stephanieae* Valdéz, 2005 (Mollusca, Gastropoda, Nudibranchia). Front Zool. Springer; 2010;7:5. 10.1186/1742-9994-7-5 PMC2822759 20205753

[42] Carroll DJ, Kempf SC (1990). Laboratory culture of the aeolid nudibranch *Berghia verrucicornis* (Mollusca, Opisthobranchia): some aspects of its development and life history. Biol Bull.

[43] Leal MC, Nunes C, Engrola S, Dinis MT, Calado R (2012). Optimization of monoclonal production of the glass anemone *Aiptasia pallida* (Agassiz in Verrill, 1864). Aquaculture.

[44] Vorobyeva OA, Malakhov VV, Ekimova IA (2021). General and fine structure of *Aeolidia papillosa* cnidosacs (Gastropoda: Nudibranchia). J Morphol.

[45] Miller JA, Byrne M (2005). Ceratal autotomy and regeneration in the aeolid nudibranch *Phidiana crassicornis* and the role of predators. Invertebr Biol.

[46] Togawa Y. Studies on Cnidophage, Specialized Cell for Kleptocnida, of *Pteraeolidia semperi* (Mollusca: Gastropoda: Nudibranchia) [PhD]. University of Tsukuba; 2021 [cited 2022 Jan 13]. Available from: https://tsukuba.repo.nii.ac.jp/records/2000808

[47] Summerton JE (2007). Morpholino, siRNA, and S-DNA compared: impact of structure and mechanism of action on off-target effects and sequence specificity. Curr Top Med Chem.

[48] Fautin DG (2009). Structural diversity, systematics, and evolution of cnidae. Toxicon.

[49] McDonald GR, Nybakken JW. List of the Worldwide Food Habits of Nudibranchs. Veliger. 1997 [cited 2022 Jan 13];40. Available from: https://escholarship.org/uc/item/0g75h1q3

[50] Krayesky SL, Mahoney JL, Kinler KM, Peltier S, Calais W, Allaire K (2010). Regulation of spirocyst discharge in the model sea anemone *Nematostella vectensis*. Mar Biol.

[51] Conklin EJ, Mariscal RN. Increase in Nematocyst and Spirocyst discharge in a sea anemone in response to mechanical stimulation. Coelenterate Ecol Behav. 1976;549–58.

[52] Garese A, García-Matucheski S, Acuña FH, Muniain C. Feeding Behavior of *Spurilla* sp. (Mollusca: Opisthobranchia) with a Description of the Kleptocnidae sequestered from its sea anemone prey. Zool Stud. 2012;51:905–12.

[53] Doumenc D (1972). Adaptation morphologique de l’acrorrhage chez *Actinia equina* L. Z Zellforsch Mikrosk Anat.

[54] Mariscal RN, Conklin EJ, Bigger CH. The ptychocyst, a major new category of cnida used in tube construction by a cerianthid anemone. Biol Bull. 1977;152:392–405.

[55] Conklin EJ, Mariscal RN (1977). Feeding behavior, ceras structure, and nematocyst storage in the Aeolid Nudibranch, *Spurilla Neapolitana* (Mollusca). Bull Mar Sci.

[56] Frick KE (2005). Nematocyst complements of nudibranchs in the genus *Flabellina* in the Gulf of Maine and the effect of diet manipulations on the cnidom of *Flabellina verrucosa*. Mar Biol.

[57] Kramer A, Francis L. Predation resistance and nematocyst scaling for *Metridium senile* and *M. farcimen*. Biol Bull. 2004;207:130–40. 10.2307/1543587 15501854

[58] Stachowicz JJ, Lindquist N (2000). Hydroid defenses against predators: the importance of secondary metabolites versus nematocysts. Oecologia.

[59] Martin R, Walther P (2002). Effects of discharging nematocysts when an eolid nudibranch feeds on a hydroid. J Mar Biol Assoc.

[60] Melo Clavijo J, Frankenbach S, Fidalgo C, Serôdio J, Donath A, Preisfeld A (2020). Identification of scavenger receptors and thrombospondin-type-1 repeat proteins potentially relevant for plastid recognition in Sacoglossa. Ecol Evol.

[61] Schindelin J, Arganda-Carreras I, Frise E, Kaynig V, Longair M, Pietzsch T (2012). Fiji: an open-source platform for biological-image analysis. Nat Methods.

[62] Mutterer J, Zinck E (2013). Quick-and-clean article figures with FigureJ. J Microsc.

[63] Szczepanek S, Cikala M, David CN. Poly-γ-glutamate synthesis during formation of nematocyst capsules in *Hydra*. J Cell Sci.; 2002;115:745–51. 10.1242/jcs.115.4.745 11865030

