# Supplementary material for: Movement and storage of nematocysts across development in the nudibranch Berghia stephanieae (Valdés, 2005)
Source: Front Zool. 2022 Apr 18;19:16. doi: 10.1186/s12983-022-00460-1 (PMC9016961; doi:10.1186/s12983-022-00460-1)
Supplement: Supplementary file 1 — Additional file 1. Supplementary Figures S1–3. [file 12983_2022_460_MOESM1_ESM.docx]

# Supplemental Figures

**
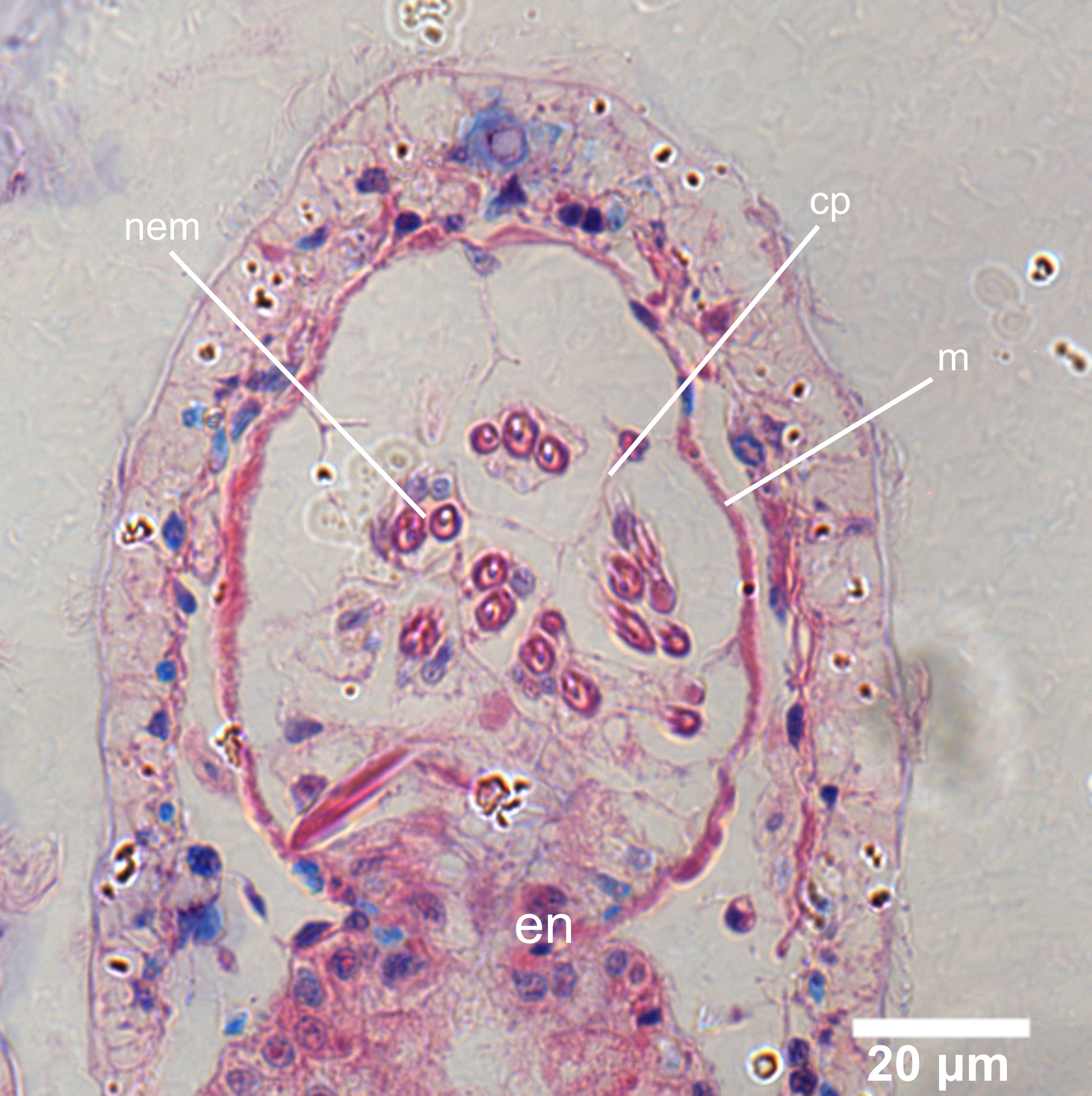
**

**Supplementary Figure 1.** Histological section of a *Berghia stephanieae* cnidosac showing a clear and simple entrance from the digestive gland to the cnidosac. Abbreviations: cp, cnidophages; en, entrance; m, musculature; nem, sequestered nematocysts.


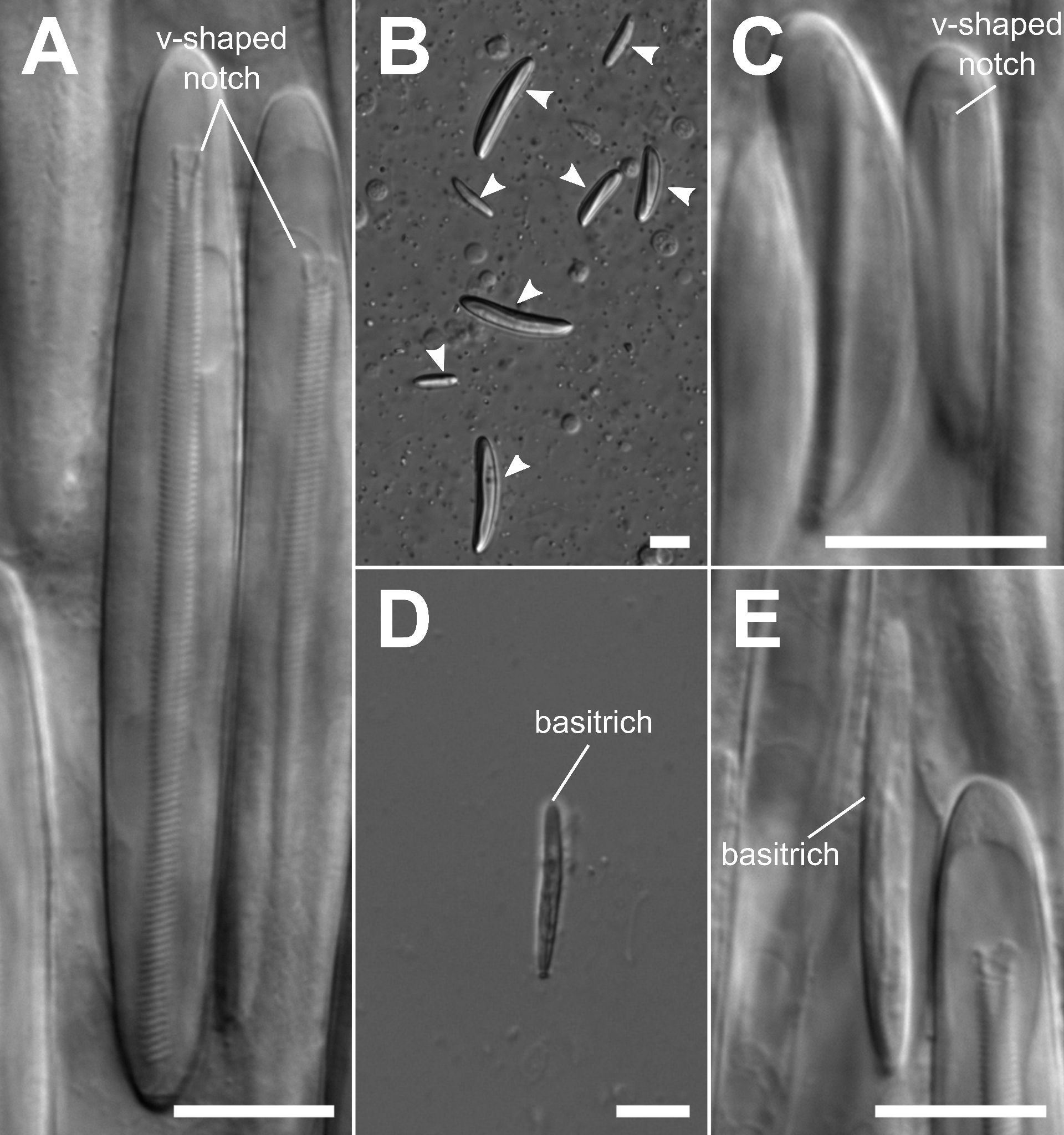


**Supplementary Figure 2.** DIC images of unfired nematocysts from inside the *Berghia stephanieae* cnidosac, including (A,C,E) nematocysts still packaged inside the cnidophage cells, and (B,D) nematocysts extruded from the cnidosac. Nematocyst types identified include: (A) large p-mastigophores from the *Exaiptasia diaphana* acontia, identified by their characteristic v-shaped notch and large size; (B) mastigophores of various sizes (arrows), including (C) small p-amastigophores (with the v-shaped notch); and (D-E) basitrichous isorhizas (basitrichs). Scale bars = 10 μm.

**
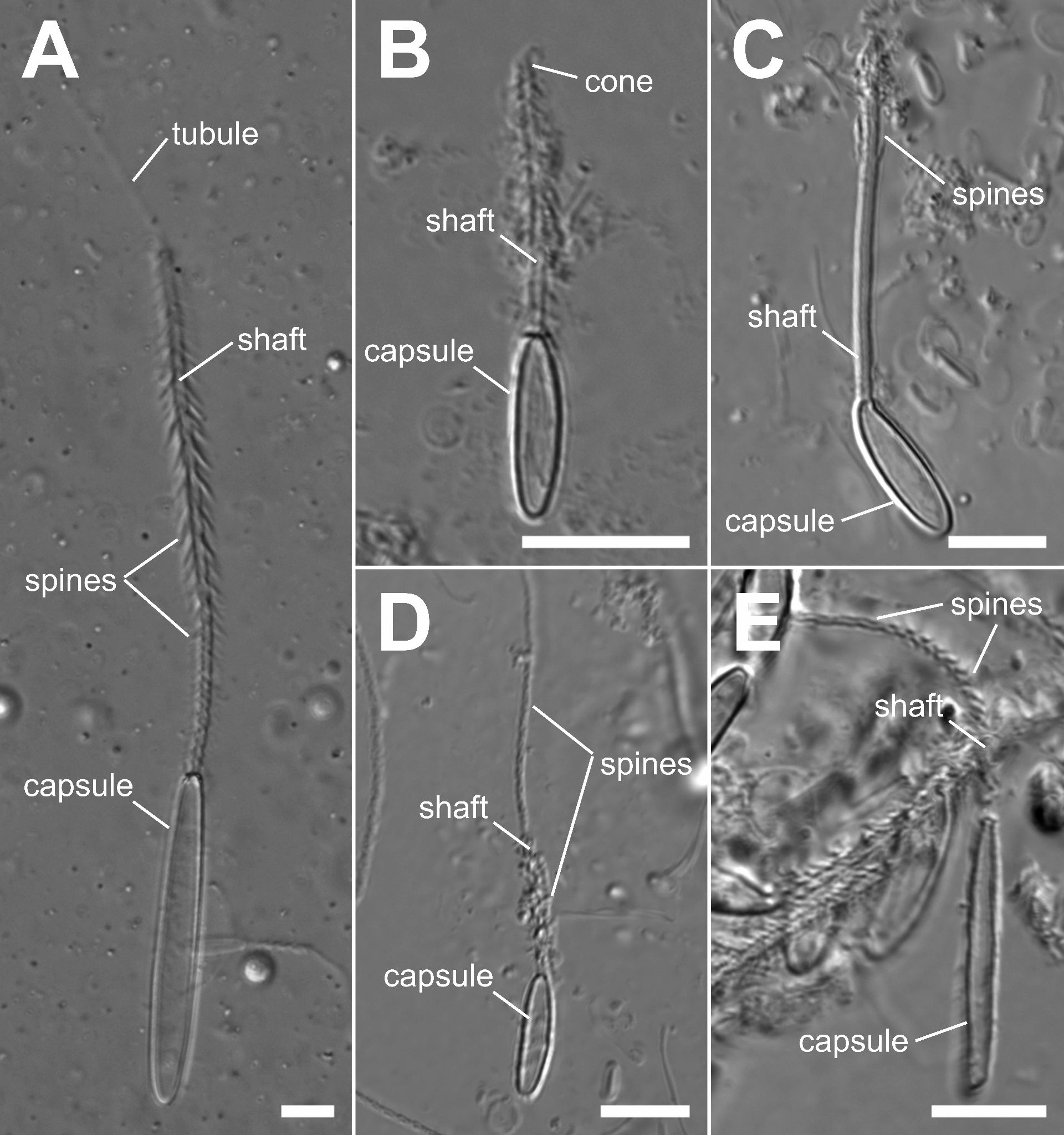
**

**Supplementary Figure 3.** DIC images of fired nematocysts from inside the *Berghia stephanieae* cnidosac. Nematocyst types identified include: (A) large p-mastigophores from the *Exaiptasia diaphana* acontia, identified by their characteristic narrow tubule extending from the end of the shaft, the increase in spine size distally along the shaft, and their large size; (B-C) smaller p-amastigophores, identified by the increase in spine size distally along the shaft, with the shaft ending in a cone; (D-E) basitrichous isorhizas identified by the large spine size along the base of the shaft, with shorter spines towards the tips. Scale bars = 10 μm.
